# Validation of pencil beam scanning proton therapy with multi‐leaf collimator calculated by a commercial Monte Carlo dose engine

**DOI:** 10.1002/acm2.13817

**Published:** 2022-11-24

**Authors:** Yuki Tominaga, Yusuke Sakurai, Junya Miyata, Shuichi Harada, Takashi Akagi, Masataka Oita

**Affiliations:** ^1^ Department of Radiotherapy, Medical Co. Hakuhokai Osaka Proton Therapy Clinic Osaka Japan; ^2^ Division of Radiological Technology Graduate School of Interdisciplinary Science and Engineering in Health Systems Okayama University Okayama Japan; ^3^ Department of Radiological technology Kurashiki Central Hospital Okayama Japan; ^4^ Hyogo Ion Beam Medical Support Hyogo Japan

**Keywords:** commissioning, lateral penumbra, multi‐leaf collimator, pencil beam scanning, proton therapy

## Abstract

This study aimed to evaluate the clinical beam commissioning results and lateral penumbra characteristics of our new pencil beam scanning (PBS) proton therapy using a multi‐leaf collimator (MLC) calculated by use of a commercial Monte Carlo dose engine. Eighteen collimated uniform dose plans for cubic targets were optimized by the RayStation 9A treatment planning system (TPS), varying scan area, modulation widths, measurement depths, and collimator angles. To test the patient‐specific measurements, we also created and verified five clinically realistic PBS plans with the MLC, such as the liver, prostate, base‐of‐skull, C‐shape, and head‐and‐neck. The verification measurements consist of the depth dose (DD), lateral profile (LP), and absolute dose (AD). We compared the LPs and ADs between the calculation and measurements. For the cubic plans, the gamma index pass rates (γ‐passing) were on average 96.5% ± 4.0% at 3%/3 mm for the DD and 95.2% ± 7.6% at 2%/2 mm for the LP. In several LP measurements less than 75 mm depths, the γ‐passing deteriorated (increased the measured doses) by less than 90% with the scattering such as the MLC edge and range shifter. The deteriorated γ‐passing was satisfied by more than 90% at 2%/2 mm using uncollimated beams instead of collimated beams except for three planes. The AD differences and the lateral penumbra width (80%–20% distance) were within ±1.9% and ± 1.1 mm, respectively. For the clinical plan measurements, the γ‐passing of LP at 2%/2 mm and the AD differences were 97.7% ± 4.2% on average and within ±1.8%, respectively. The measurements were in good agreement with the calculations of both the cubic and clinical plans inserted in the MLC except for LPs less than 75 mm regions of some cubic and clinical plans. The calculation errors in collimated beams can be mitigated by substituting uncollimated beams.

## 1 INTRODUCTION

Proton beam therapy has excellent depth dose (DD) physical properties, which enable comparable target dose conformity and lower dose around normal tissues than conventional radiotherapy.^1^ Currently, pencil beam scanning proton (PBS) therapy is the most advanced proton therapy, which uses an inverse‐planned mono‐energetic pencil beam (PB) that scans the target across the lateral plane.^2^ The scanned beams also change from high to low energies, creating a dose distribution in the depth direction.^3^ PBS enables better dose conformity in the target and reduces the surrounding organs at risk (OARs) compared to both passive scattering and uniform scanning methods.^4^, ^5^

However, current PBS accelerators can irradiate only up to approximately 70 MeV (approximately 40 mm depth in water).^6^ To irradiate superficial regions, we must insert an energy absorber called a range shifter (RS), which expands the lateral fall‐off (penumbra) by beam scattering and deteriorates the lateral dose distribution.^7^ Although PBS can also avoid critical organs with a distal fall‐off, some dose errors are considered, such as range uncertainties and increasing relative biological effectiveness (RBE).^8^ To overcome these issues, PBS plans are often preferred for lateral beam angle selection with respect to the location of critical organs. Thus, the PBS beam requires the lateral penumbra to be as steep as possible. Several authors have reported new techniques for improving the penumbra, such as advanced optimizing spot placements,^9^ smaller initial beam size machines,^10^ and patient‐specific aperture systems.^11^, ^12^ Furthermore, the calculated dose by increasing the distance between the patient and RS (called air gap) deteriorates the penumbra and causes dose discrepancy due to the nuclear halo.^13^ To mitigate these effects, the PBS beam should be irradiated with RS as close as possible to the patient's surface.

Our PBS proton therapy system, which has a multi‐leaf collimator (MLC) downstream of a movable nozzle, could be irradiated to patients as close as possible. This machine is the same as the one used in the studies by Fukumitsu and Sugiyama et al.^14^, ^15^ By using the MLC, no additional collimation hardware is required for each beam, thus improving the treatment throughput and reducing the risk of dropping patients. Our commercial treatment planning system (TPS) could also perform Monte Carlo (MC) dose calculations,^16^, ^17^ thus reducing the above mentioned problem of dose discrepancy with an increasing air gap. Although several reports have published planning simulations and commissioning results about using an MLC,^14^, ^15^, ^18^, ^19^, ^20^ no report was found on the TPS validations of this PBS system with the MLC. Here, this study aimed to validate the clinical beam commissioning using the MLC for cubic target volumes of various sizes and clinically realistic plans. We first evaluated the effectiveness of lateral penumbra with static collimators in anticipation of future updates to the dynamic collimation technique.

## 2 METHODS

### 2.1 Proton beam therapy system

**FIGURE 1:**
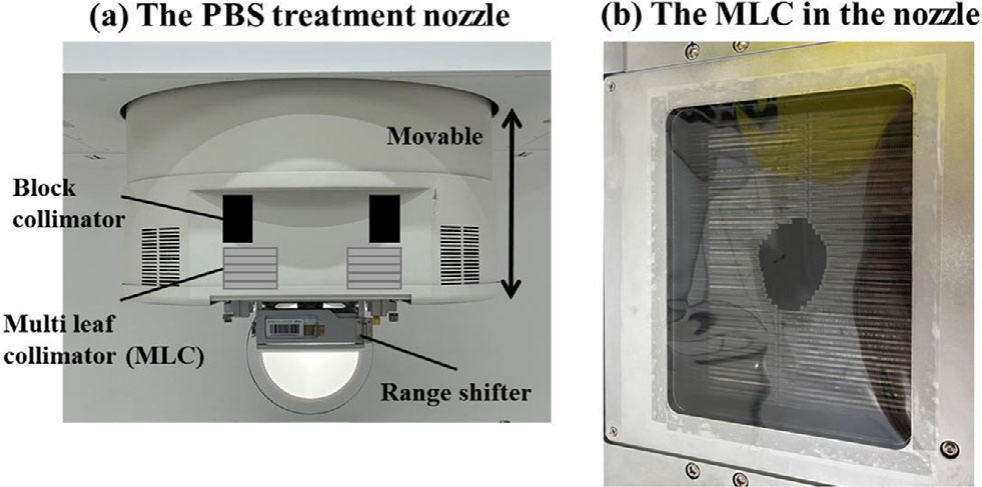
The schematic and image of (a) our PBS treatment nozzle with the multi‐leaf collimator (MLC) and (b) the MLC system in the nozzle

The Medical Corporation, Osaka Proton Therapy Clinic, has a MELTHEA V (Hitachi, Ltd., Tokyo, Japan) proton beam system. This system has a synchrotron and gantry room with a selected beam nozzle (Figure 1). The users could select both lattice scanning and PBS for raster scanning for each patient. The nozzle with the MLC on the tip could approach the isocenter by a minimum of 250 mm, thus the PBS beam could irradiate with RS and MLC as close as possible to the patient's surface. Depending on the policy of treatment planning, the PBS system can irradiate both collimated and uncollimated beams in the planning phase. The MLC is used externally from the target by an arbitrary margin. The MLC is positioned stationary outside when all energy layer spots are irradiated. Although the spots and MLC may overlap depending on the distance between the collimator and the target volume, the resulting dose distribution of collimated (cut) spot are also optimized by the TPS. Since the leaf positions can be manually changed, it is possible to hide the specific OAR region from the direction of the beam using several leaves to spare OARs such as the passive scattering method. This PBS system has 70.7–235.0 MeV energies and is available for using seven polyethylene RSs 60–66 mm water‐equivalent thickness (WET) downstream of the nozzle to irradiate less than 40 mm. The MLC consists of 54 pairs of leaves and is made of iron with a thickness of 140 mm and a leaf width of 3.75 mm. Individual leaves can extend up to 75 mm in the lateral direction, resulting in a maximum collimator open size of 150 × 200 mm^2^. The distance between the isocenter and MLC varied from 250 to 560 mm, and the maximum field area at the isocenter ranged from 162 × 216 to 184 × 246 mm^2^. More information about this machine can be found in the paper by Fukumitsu et al.^14^

### 2.2 TPS validation

The RayStation 9A TPS (RaySearch, Stockholm, Sweden) has a commercial MC dose calculation engine.^21^ The MC must be used for all collimated beams to consider scattering from both the MLC and RS. According to the TPS reference manual, the modeling for the nuclear halo was calculated based on two papers by Soukup et al. and Pedroni et al. in 2005.^22^, ^23^ These works reported that the width and relative weight of the nuclear scattering Gaussian is calculated as a function of the initial beam energy and the radiological depth. To consider the dose contribution of nuclear interaction, a second ‘nuclear’ pencil beam is used in addition to the PB whose transverse spread is described by the multiple scattering.

**FIGURE 2:**
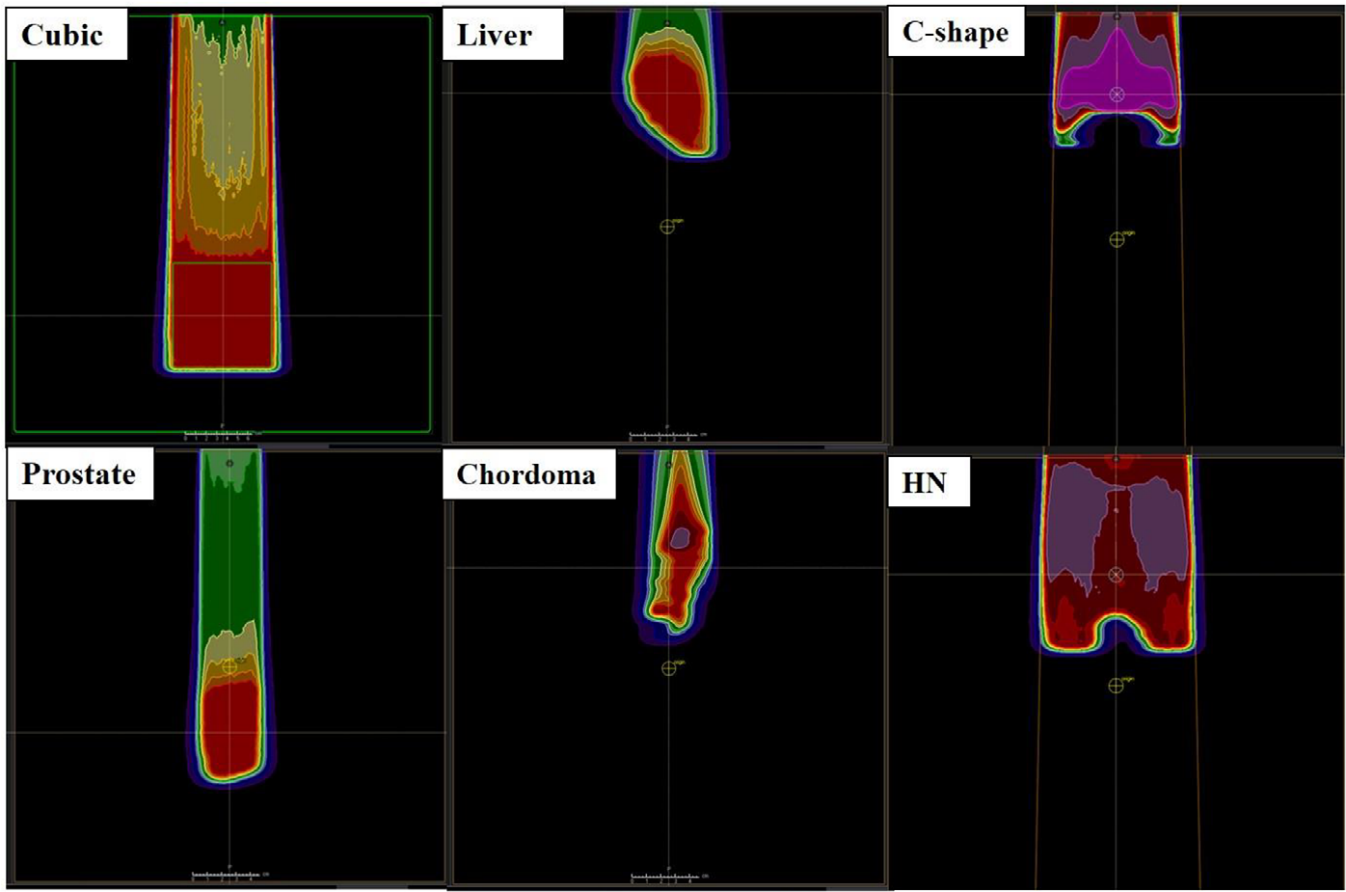
The screenshots of verification plans in a water phantom for a cubic target and five clinical cases

Eighteen verification plans for cubic targets were created with field sizes of 40 mm × 40 mm^2^ and 95 mm × 95 mm^2^, various beam ranges, and spread‐out Bragg peak (SOBP) widths of 90 mm. All plans consisted of a single field optimized (SFO) beam with a gantry angle of 0°, and the isocenter was determined at the center of the SOBP. The dose calculation grid was 2.0 × 2.0 × 2.0 mm^3^ for all plans. An RS of 60 mm was used for 9 of the 18 plans, and the three types of 45° snout rotated beams were included for both the RS and without RS plans. The air gaps were set to 150 mm, except for the three shallowest plans without RS (air gap of 164 mm). To cover each target adequately, the MLC leaf margins were determined at 1.4–7.2 mm with respect to each target. The prescribed doses covered 1.0 GyRBE with 98% of each target volume. Table 1 shows the beam parameters for 18 cubic plans. Figure 2 shows a screenshot in the TPS for the representative cubic plan (called Cubic 1 in Table 1).

**TABLE 1:** Beam parameters for 18 cubic and fourteen clinical verification plans and the middle region (ranges of all planes each plan) γ‐passing of lateral profiles at 2%/2 mm. The single‐field optimized (SFO) and multi‐field optimization (MFO) mean single and multi‐field optimizations, respectively

Beam name	Gantry (°)	Snout (°)	Isocenter (mm)	Range (mm)	RS (mm)	Number of layers	Air gap (mm)	MLC (mm)	Optimize pattern*	Scan pattern	Middle (ranges) gamma passing of lateral profiles at 2%/2 mm (%)
Cubic 1	0	0	288	341.9	None	18	150	5.1	PB‐MC	SFO	99.4 (73.7–99.4)
Cubic 2	0	0	288	341.9	None	18	150	7.2	PB‐MC	SFO	97.9 (95.9–97.9)
Cubic 3	0	0	185	241.3	None	25	150	3.1	PB‐MC	SFO	99.2 (63.0–99.2)
Cubic 4	0	0	185	241.3	None	25	150	6.1	PB‐MC	SFO	100 (92.0–100.0)
Cubic 5	0	0	86	141.5	None	52	164	1.4	PB‐MC	SFO	96.7 (81.8–99.2)
Cubic 6	0	0	86	141.5	None	52	164	5.3	PB‐MC	SFO	100 (97.4–100.0)
Cubic 7	0	45	288	341.9	None	18	150	4.0	PB‐MC	SFO	100 (87.2–100.0)
Cubic 8	0	45	185	241.3	None	25	150	5.0	PB‐MC	SFO	100 (71.9–100.0)
Cubic 9	0	45	86	141.5	None	52	164	5.0	PB‐MC	SFO	94.2 (83.5–99.3)
Cubic 10	0	0	227	284.4	60.0	18	150	6.4	PB‐MC	SFO	99.4 (89.3–99.4)
Cubic 11	0	0	227	284.4	60.0	18	150	3.3	PB‐MC	SFO	95.6 (93.8–100.0)
Cubic 12	0	0	135	189	60.0	22	150	4.5	PB‐MC	SFO	98.8 (96.6–99.4)
Cubic 13	0	0	135	189	60.0	22	150	6.8	PB‐MC	SFO	100 (100.0–100.0)
Cubic 14	0	0	45	98	60.0	36	150	6.9	PB‐MC	SFO	93.3 (72.0‐100.0)
Cubic 15	0	0	45	98	60.0	36	150	5.9	PB‐MC	SFO	97.8 (91.1–97.8)
Cubic 16	0	45	227	284.4	60.0	18	150	5.0	PB‐MC	SFO	99.4 (90.1–99.4)
Cubic 17	0	45	135	189	60.0	22	150	4.5	PB‐MC	SFO	100 (100.0–100.0)
Cubic 18	0	45	45	98	60.0	36	150	5.0	PB‐MC	SFO	100 (96.4–100.0)
Liver 1	30	0	57	85.0	61.0	24	109.2	13.0	MC‐MC	SFO	100 (100.0–100.0)
Liver 2	315	0	36	65.0	60.0	23	116.4	13.0	MC‐MC	SFO	100 (100.0–100.0)
Liver 3	270	0	51	78.5	60.0	25	99.2	13.0	MC‐MC	SFO	100 (100.0–100.0)
Prostate 1	90	0	196	236.3	None	17	160.7	9.0	PB‐MC	SFO	100 (100.0–100.0)
Prostate 2	270	0	199	241.3	None	17	157.5	9.0	PB‐MC	SFO	100 (97.4–100.0)
Chordoma 1	280	0	80	139.6	2.0	38	148.0	8.0	MC‐MC	MFO	99 (90.2–99.0)
Chordoma 2	80	0	80	138.2	None	43	146.7	8.0	MC‐MC	MFO	92.8 (91.8–100.0)
Chordoma 3	180	0	150	194.7	2.0	21	135.8	8.0	MC‐MC	MFO	99 (98.0–100.0)
C‐shape 1	0	0	51	88.5	None	34	199.2	5.0	MC‐MC	MFO	96.8 (96.8–100.0)
C‐shape 2	90	0	127	192.3	None	22	99.7	5.0	MC‐MC	MFO	100 (100.0–100.0)
C‐shape 3	270	0	127	192.3	None	22	100.1	5.0	MC‐MC	MFO	100 (90.9–100.0)
HN 1	0	0	75	129.9	60.0	34	57.4	5.0	MC‐MC	MFO	86.2 (86.2–98.6)
HN 2	150	0	87	162.4	60.0	36	93.1	5.0	MC‐MC	MFO	98.7 (95.5–100.0)
HN 3	210	0	87	162.4	60.0	37	94.2	5.0	MC‐MC	MFO	98.7 (81.7–100.0)

* Abbreviations: PB‐MC, Pencil beam algorithm optimization followed by Monte Carlo (MC) dose calculation; MC‐MC, MC optimization followed by MC dose calculation.

### 2.3 Verification plans

To evaluate patient‐specific TPS validation, we selected three representative clinical cases and the C‐shape and head and neck (HN) phantoms of the American Association of Physicists in Medicine (AAPM) Task Group Report 119 (TG‐119).^24^ Lower part of Table 1 shows the beam parameters of five clinical realistic plans. The three clinical cases consisted of liver recurrence of intrahepatic cholangiocarcinoma (liver), prostate cancer (prostate), and skull base chordoma (chordoma). The selected patient information was approved by the institutional review board at Osaka Proton Therapy Clinic (No. 20201016‐2). All helical CT scans were acquired in the supine position using an Aquillion LB TSX 201A (Canon, Tokyo, Japan). Since constant respiratory movements were observed in the liver, gated CT scans were acquired using AZ‐733 V (Anzai, Tokyo, Japan). The gross tumor volumes (GTVs) and several surrounding OARs were delineated by an experienced oncologist at each site. The clinical target volumes (CTVs) were created by expanding from the GTVs by 5 mm for the liver and chordoma cases excluding the anatomical barriers. The planning target volumes (PTVs) were geometrically expanded from the CTVs by 5 mm for the prostate and 3 mm for the chordoma. For the liver case, the internal target volume (ITV) was expanded by 1–2 mm from the CTV to consider respiratory motion, and the PTV was expanded from the ITV by 5 mm.

The dose prescriptions covered 66 GyRBE in 10 fractions for the liver, 63 GyRBE in 21 fractions for the prostate, and 70 GyRBE in 35 fractions for chordoma, with 50% of each CTV. The beam angles were selected to maintain target coverage and minimize OAR doses; the number of fields was determined with three for the liver and chordoma and two opposite fields for the prostate. Due to the automatic setting for TPS optimization, the RSs of 60–61 mm for the liver and 2 mm for the chordoma were used, and no RS was used for the prostate case. Worst‐case optimization was used with setup uncertainty for six directions of ± 5 mm for both the liver and prostate, and ±3 mm for the chordoma.^25^ An additional ±3.5% range uncertainties were added to all cases.^26^ Spot optimization techniques were SFO for the liver and prostate and multi‐field optimization (MFO) for chordoma. The spot spacing of all the energy layers was one spot sigma multiplied by 0.8 in water for all plans. To ensure the robustness of the CTV, the MLC was collimated with a margin of 13.0 mm for the Liver, 9.0 mm for the prostate, and 8.0 mm for the Chordoma with respect to each CTV. All plans were optimized on a 2 mm calculation grid. Although the TPS could optimize both the PB and the MC algorithms, each optimization algorithm was determined to be the same as our clinic conditions.^27^ The liver and chordoma plans were optimized using the MC algorithm with a sampling history of 5000 ions/spot, while the prostate plan was optimized using the PB algorithm. Normally, the PBS plans were optimized by the MC algorithm; thus, we use the PB algorithm for prostate plans in plan optimization of clinical use to save planning time. The final dose calculation used the MC algorithm with a 0.5% statistical uncertainty for all plans.

The C‐shape and HN plans were created according to the dose constraints presented in the TG‐119 with three fields in both plans. The optimization algorithm, statistical uncertainty, and calculation grid were used at 5000 ions/spot, 0.5%, and 2.0 mm, respectively. The C‐shape plan was satisfied with the hard constraints (10% volume to receive less than 10 GyRBE) of the core OAR and 95% of the PTV to receive 50 GyRBE (ref 24 in Table 6^24^). The HN plan could satisfy all dose constraints of the targets and OARs in the Table 6.^24^ The 14 collimated beams of the five verification plans were copied to the same water phantom used to calculate the cubic plan. The WET from the patient surface to the isocenter was determined for each isocenter. Figure 2 shows the screenshots in the TPS for each first beam of the five clinical verification plans. The cross line in the figure indicates the isocenters of each plan.

### 2.4 Evaluation tools

**FIGURE 3:**
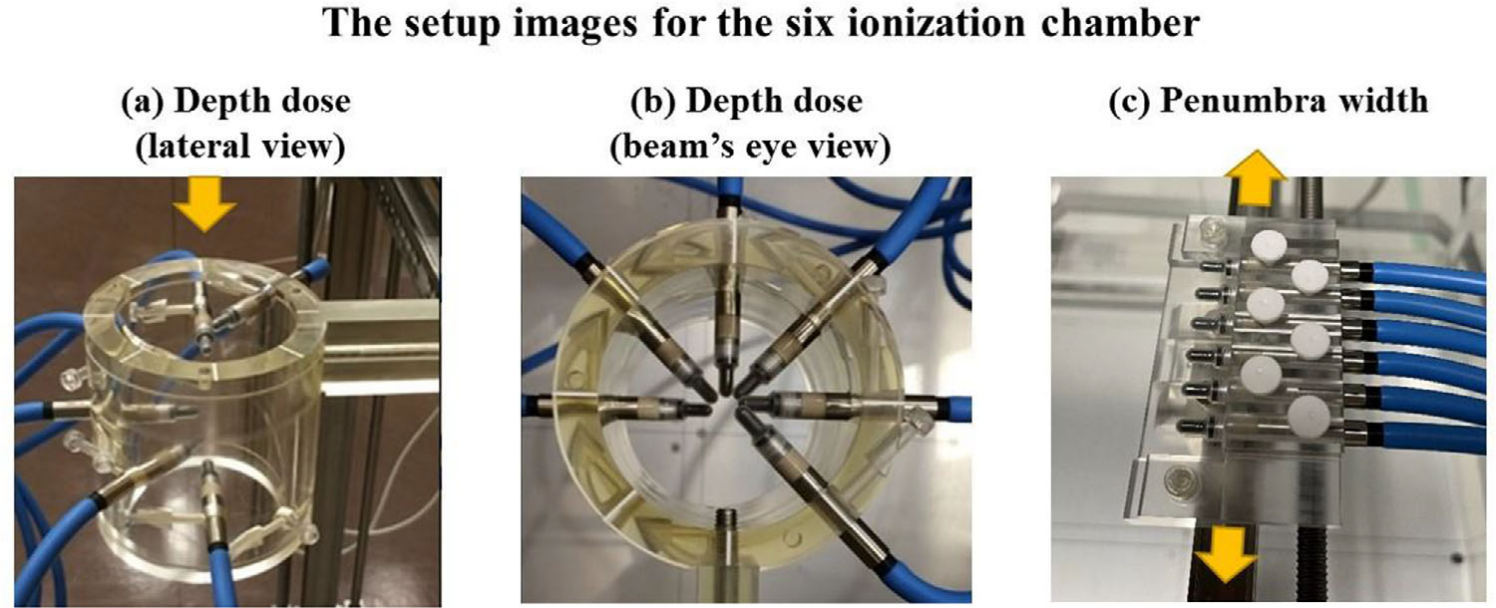
The setup images for the six PinPoint ionization chambers for the (a) lateral view of the depth dose, (b) beam's eye view of the depth dose, and (c) lateral penumbra. The distance between the chambers for both attachments is 10 mm

Beam measurements were performed for the DDs, lateral penumbra widths at the isocenter, lateral profiles (LPs), and absolute doses (ADs) at the isocenter with a gantry angle of 0°. Both DDs and lateral penumbra used the 3‐dimensional (3D) water phantom (MP3‐M; PTW Freiburg, Germany), six PinPoint ionization chambers (Type 31015; PTW), a multi‐channel electrometer (MULTIDOS; PTW), and a measurement control software (HRD; HIBMS, Hyogo, Japan). As shown in Figure 3, the measurement attachments of DDs and lateral penumbra are arranged in helical and lateral directions, and each chamber is positioned at 10 mm intervals. The six chambers were arranged so that the chamber hit the dedicated pillar placed in the central axis for DD attachment. Then, a mono‐energetic beam (141.2 MeV) was used to check the beam range agreement after setting both DD and lateral penumbra attachments. The cross‐calibration between the PinPoint chamber used for port 1 of the MULTIDOS and a calibrated Semiflex ionization chamber (Type 31013; PTW) was measured in the mono‐energetic (141.2 MeV) uniform fields of 102 × 102 mm^2^ at a depth of 25 mm. Then, the correction coefficients between the charge of the port 1 chamber and the other chambers were also measured under the same mono‐energetic fields and was registered in the MULTIDOS console as the calibration value. Twelve DDs were measured, except for collimator‐rotated beams, which were analyzed using the gamma index at the criteria of 3%/3 mm and a dose threshold above 10% of the maximum dose.^28^ Then, the lateral penumbra widths (defined as the distance between 80% and 20% with respect to isodose points) at the central axis were measured for all plans in both the x (left‐right) and y (superior‐inferior) directions at the isocenter.^29^ The penumbra widths were compared between the measured values and TPS calculations.

The ADs were measured at the center of the SOBP for all 18 plans, which was the same as the isocenter. Absolute dose measurements were performed using the solid water phantom (Tough Water; Kyoto Kagaku, Kyoto, Japan) and a PinPoint 3D ion chamber (Type 31022; PTW). The Tough Water has a hole in the isocenter for inserting the 3D PinPoint chamber and is installed so that blocks corresponding to the measurement depth are stacked. The measured physical doses were scaled up by a factor of 1.1 to convert the equivalent doses to account for RBE in the TPS. Dose differences (δ) were calculated between the measured doses (D_meas_) and the calculated doses (D_calc_) using Equation (1).

(1) $$\delta =100\times \;\frac{\left(D_{meas}\;-\;D_{calc}\right)}{D_{calc}}\;\left(\%\right)$$

Lateral dose profiles were measured using a 2D ionization chamber array (2D‐Array, OCTAVIUS 729 XDR; PTW) and the Tough Water. The 27 × 27 array chambers are spaced 10 mm apart from center to center of each chamber. Each chamber size is 5 × 5 × 3 mm^3^ and the chamber volume is 0.075 cm^3^. Four lateral dose profiles of each SOBP were measured at the plateau (25 mm), proximal, middle, and distal planes. The proximal and distal planes were measured 5.0 mm inside from the 95% SOBP edge in the isocenter direction. The total measured LPs consisted of 69 planes for the 18 plans. Measured profiles were analyzed using the gamma index at the criteria of 2%/2 mm and 3%/3 mm with a dose threshold of 10% with respect to the maximum dose.

For patient‐specific TPS validation, we measured the LPs at the three depths (proximal, middle, and distal) and the ADs at the isocenter for the 14 collimated beams. This measuring device was the same as that used for the dose verification of the cubic plan. Measured profiles were analyzed using the gamma index at the criteria of 2%/2 mm and 3%/3 mm, and the ADs were compared between the measured and calculated values.

## 3 RESULTS

### 3.1 Collimated beam measurements

**FIGURE 4:**
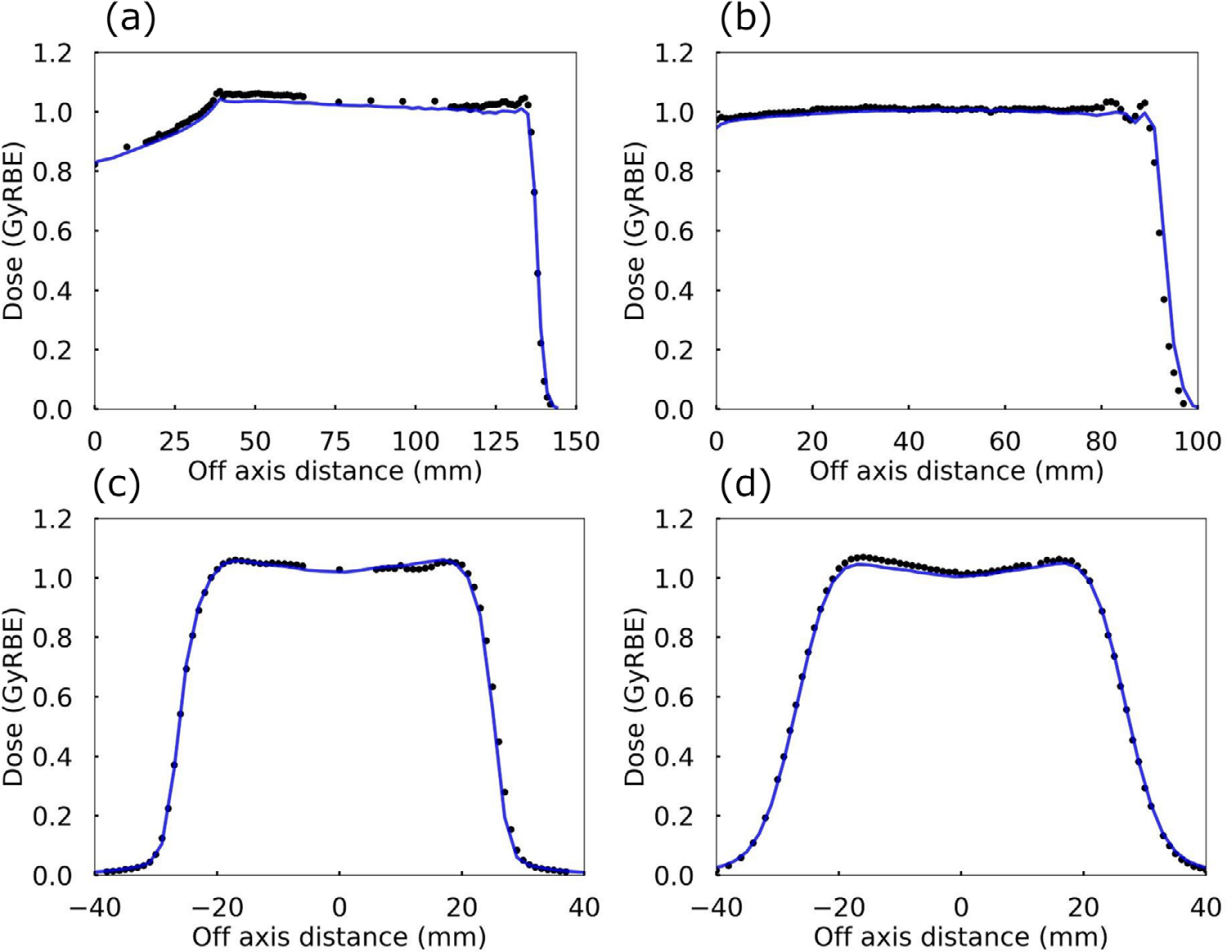
Comparison of measured (plots) and calculated (solid) doses, the upper row shows the representative depth doses and the lower row shows the lateral profiles at the isocenter. The left columns show the collimated beam without RS, and the right columns show the collimated beam with RS

Figure 4 shows two representative collimated beam results for the measured (plots) and calculated (solid lines) doses. Figure 4a,b shows the DDs both without RS (beam ranges of 135 mm and field width of 40 mm) and with RS (beam ranges of 90 mm and field width of 40 mm). Figure 4c and d show the lateral penumbra measurements (x planes) at the isocenter in the cross‐section of Figure 4a,b. The average ± standard deviation (ranges) gamma index pass rates (γ‐passing) at 3%/3 mm of the 12 DDs were 96.5 ± 4.0% (89.7%–100.0%). The γ‐passing of 69 LPs was on average 95.2 ± 7.6% (63.0%–100.0%) at 2%/2 mm and 98.8 ± 2.9% (87.4%–100.0%) at 3%/3 mm, respectively. The AD differences at the isocenter were, on average, 0.7 ± 0.9% (−1.9%–1.8%). The lateral penumbra width differences were, on average, 0.4 ± 0.3 mm (0.1–1.1 mm) for the x‐planes and 0.3 ± 0.2 mm (0.1–0.7 mm) for the y‐planes. The penumbra width differences of the x‐planes were within ± 1.0 mm except for a plane, and that of the y‐planes was ± 0.7 mm.

### 3.2 Patient‐specific measurements

**FIGURE 5:**
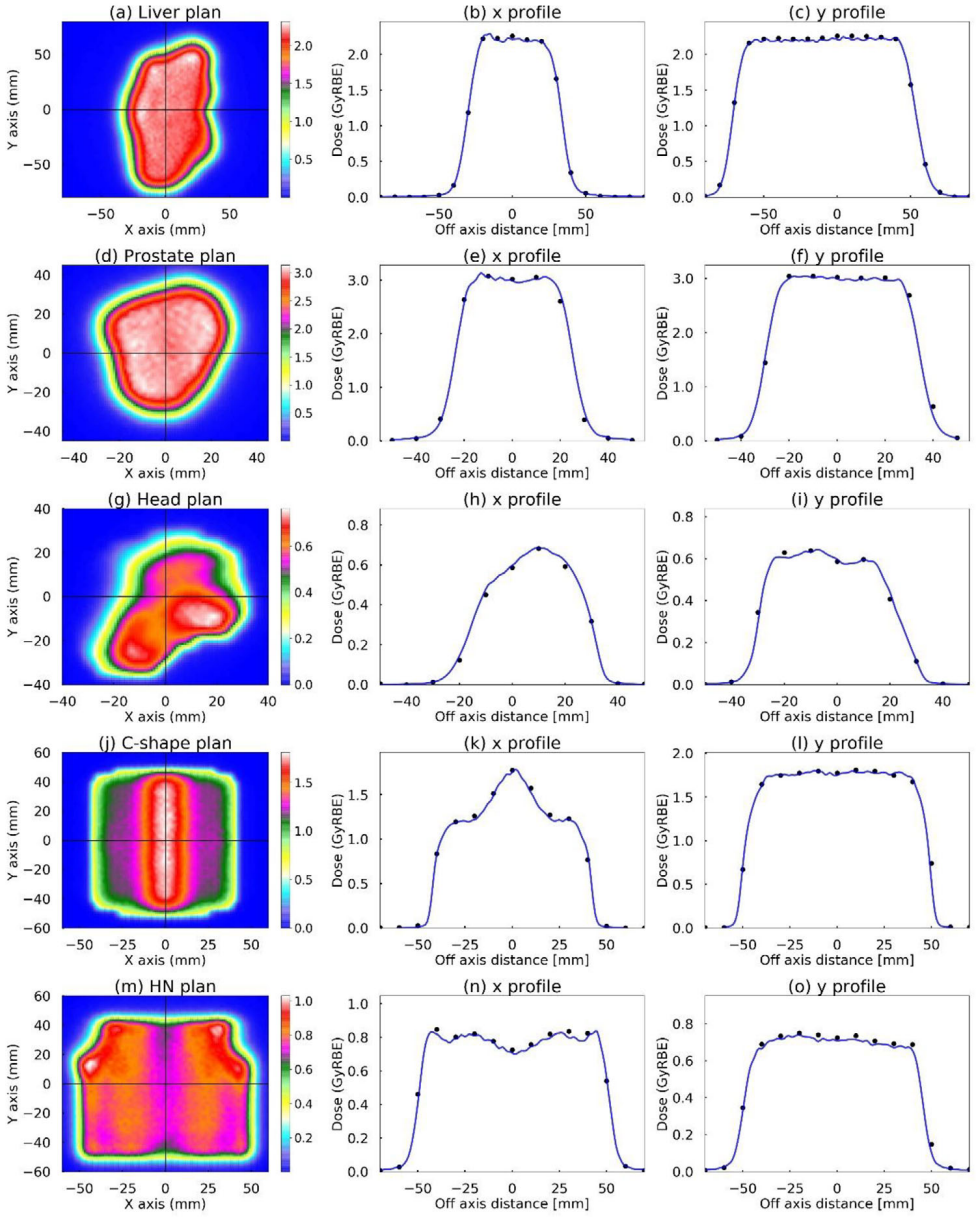
Patient‐specific verification comparison between the measured (black circles) and calculated (blue solid line) doses. The dose distribution for the coronal plane at the isocenter (left columns), the lateral profiles in the x‐direction (middle columns), and the lateral profiles in the y‐direction (right columns). The crosshairs on the dose distributions show the cross‐plane of the lateral profiles

Figure 5 shows the TPS validation results for each first beam of the five clinical cases between the measurements and calculations. The γ‐passing of 24 LPs were, on average, 97.7% ± 4.2% (81.7%–100.0%) at 2%/2 mm and 99.9% ± 0.4% (98.4%–100.0%) at 3%/3 mm, respectively. The AD differences at the isocenter were, on average, −0.2% ± 1.1% (−1.8%–1.8%). The AD differences were within ±1.2% for the SFO plans (the prostate and liver plans) and within ±1.8% for the MFO plan (the chordoma, C‐shape, and HN plans). Although the AD difference of the MFO plan was slightly higher than that of the SFO plans, all plans were in good agreement between the measurements and calculations.

**FIGURE 6:**
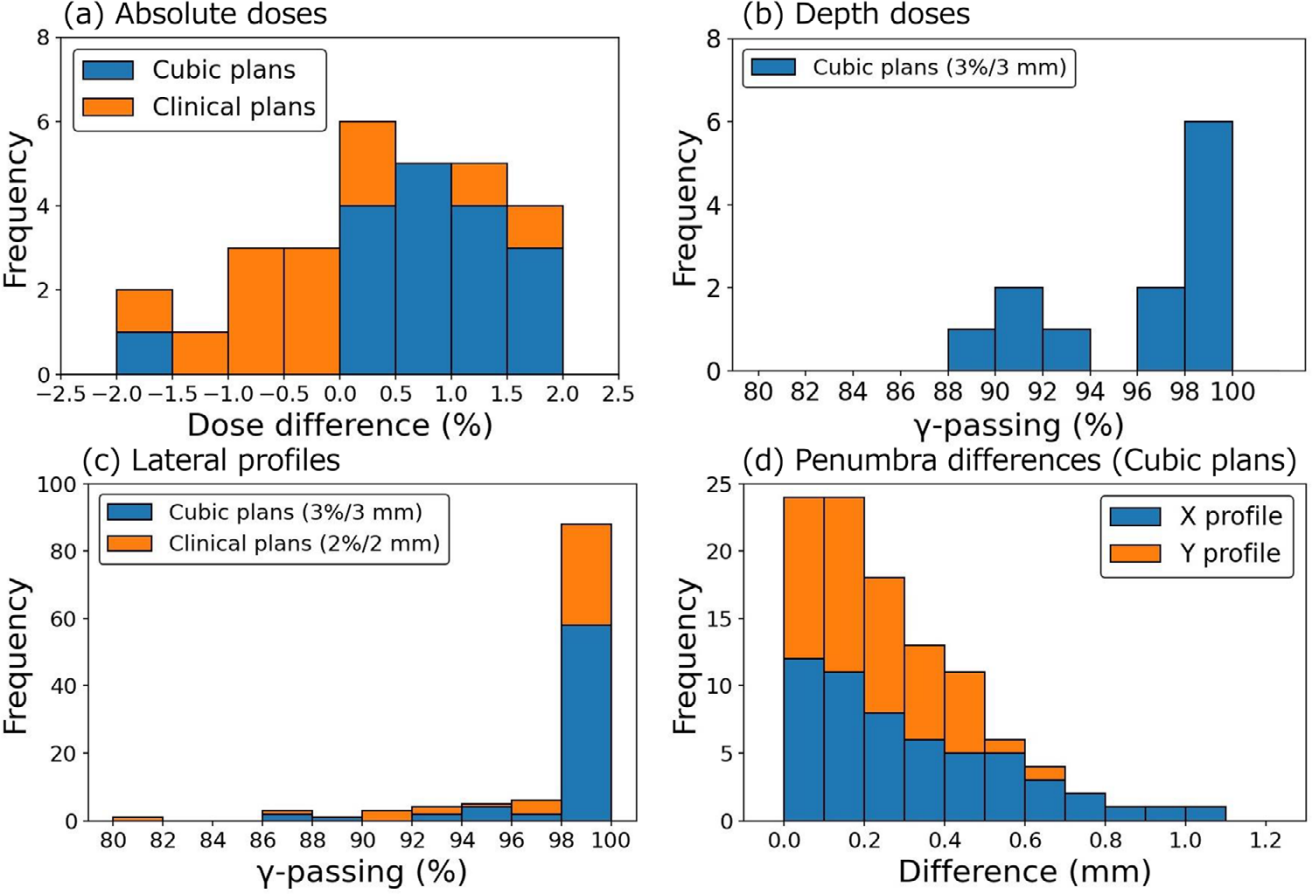
The histograms of (a) absolute dose differences for 18 cubic plans and 14 clinical plan's beams, (b) γ‐passing of depth doses for 12 cubic plans, (c) γ‐passing of lateral profiles for cubic plans at 3%/3 mm (69 planes) and clinical plans at 2%/2 mm (42 planes), and (d) lateral penumbra width differences for x and y profiles

**FIGURE 7:**
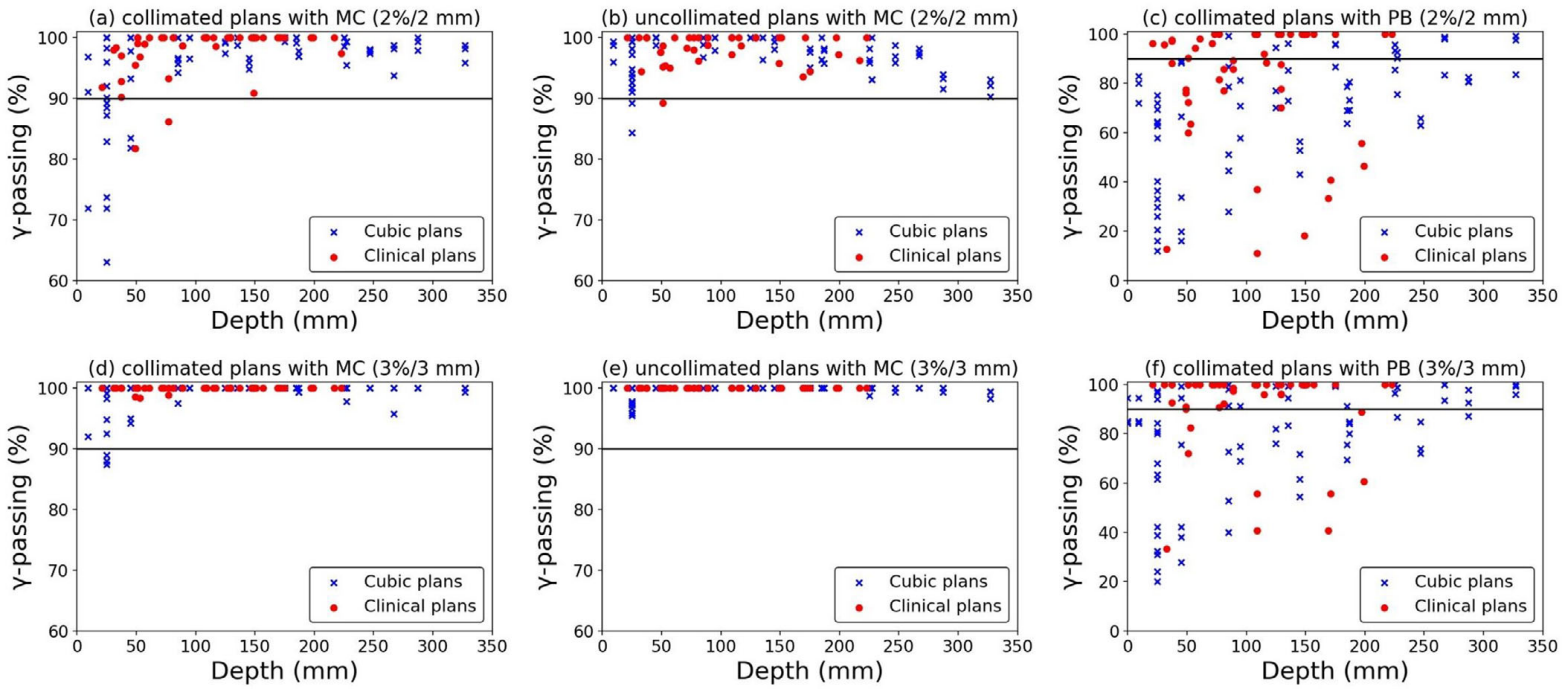
The scatter plots of lateral profile γ‐passing versus measured depth for the (a, d) collimated plans with MC, (b, e) uncollimated plans with MC, and (c, e) collimated plans with PB. The blue and red scatters show the 69 cubic and 42 clinical plan's measured planes

Figure 6a–d shows the histograms of the TPS validation results for AD differences, γ‐passing of DDs and LPs, and lateral penumbra width differences of x and y profiles for cubic plans and clinical plan beams. Figure 7 also shows the scatter plots of LP γ‐passing versus measured depth at (a) 2%/2 mm and (d) 3%/3 mm for the cubic and clinical plans. In several cubic plan's LP measurements without RS, the γ‐passing at 3%/3 mm were deteriorated (increased the measured doses). Three underestimated calculated doses in the plateau region were observed, and the γ‐passing was less than 90%. Besides, the γ‐passing were deteriorated below 90% at 2%/2 mm for 12 measured planes less than 75 mm depths both with and without RS plans (Table 1). To investigate the cause of the worsened γ‐passing at such shallow depths, we measured the LPs and analyzed for all cubic and clinical plans by excluding the MLC. Furthermore, to verify the usefulness of MC calculations for collimated PBS plans, the gamma analyses were performed between the measured LPs and the PBS plans that were calculated using the PB algorithm. The γ‐passing of uncollimated MC plans were on average 96.9% ± 3.4% (84.3%–100.0%) at 2%/2 mm and 99.6 ± 1.1% (95.6%–100.0%) at 3%/3 mm for the cubic plans and 98.3 ± 2.5% (89.3%–100.0%) at 2%/2 mm and all 100% at 3%/3 mm for the clinical plans (Figure 7b,e). Then, the γ‐passing of collimated PB plans were on average 68.4 ± 23.9% (12.0%–99.4%) at 2%/2 mm and 78.7% ± 22.5% (20.0%–100.0%) at 3%/3 mm for the cubic plans and 78.4 ± 26.0% (11.1%–100.0%) at 2%/2 mm and 89.6% ± 18.6% (33.3%–100.0%) at 3%/3 mm for the clinical plans, respectively (Figure 7c,f). Although the number of measured planes with worse γ‐passing (< 90% at 2%/2 mm) at depths below 75 mm was reduced for the uncollimated plans than those for the collimated plans, only three planes were showed with less than 90% of γ‐passing. The collimated plans with PB showed the degraded γ‐passing than these of MC at a wide depth range, confirming its unsuitability for clinical use.

## 4 DISCUSSION

We performed clinical beam commissioning of the PBS plans with the MLC and evaluated whether the penumbra regions in the collimated beams were consistent between the measurements and calculations. We also verified the end‐to‐end test and patient‐specific measurements for five collimated clinical plans.

The γ‐passing at 2%/2 mm of the LPs for both cubic plans and clinical plans were on average more than 95.0%. Most measurements were satisfied the 95.0% γ‐passing at 2%/2 mm for cubic plans and 3%/3 mm for clinical plans recommended by the AAPM TG‐185.^30^ However, the γ‐passing deteriorated (less than 90% at 2%/2 mm and 3%/3 mm) in the region of some plans less than 75 mm. We understand that the cause of the discrepancy between the measurements and calculations could be either the measurement error or the TPS dose calculation error. If this is due to measurement errors in the 2D‐Array, it should be possible to see a deterioration in the γ‐passing at other depths as well. Thus, we suspected the TPS modeling error rather than the measurement error. According to the TPS reference manual, the calculations on our PBS machine can be overestimated by nuclear halo if an RS is inserted, and by edge scattering if a collimator is inserted, which may change the dose calculation.^21^ Unfortunately, no previous reports on PBS systems with different collimation types have been indicated that the TPS showing modeling errors in these areas.^31^, ^32^, ^33^ Some of the protons near the edge of the aperture are incident on the aperture, then scatter out of the aperture again. This effect called the edge scattering effect, adds a characteristic angle to the near surface transverse dose profile, may also add dose to more central or deeper parts of the target.^21^ Since several points showed the worsen γ‐passing even in uncollimated plans, the factors affecting the dose calculation error might be due to the potential limitations of evaluation of beam scattering from the MLC and the beam modeling of the TPS. The point AD differences were within ± 1.9% for the 18 cubic plans and ±1.8% for the five clinical plans. These dose agreements satisfied the tolerance of within ±2.0% in the study commissioned by the same TPS of other PBS machines.^34^ In addition, several reports evaluated other PBS machines that calculated the same TPS and showed that the ADs were both within ±3.0% for cubic plans of various sizes and mock clinical cases.^32^, ^33^ Since our point AD results showed a similar tendency to those reports, we judged that it was acceptable for clinical use when considered in conjunction with the other commissioning results. However, it should be noted that the use of collimators in shallow depth plans because in some beams with a depth of less than 75 mm the γ‐passing of LPs may be less than 90%. If some beams have significant dose discrepancies from patient‐specific measurements, they can be appropriately avoided by interventions such as revising the treatment beam angle or using uncollimated beams. Our lateral penumbra width differences were within ±1.1 mm. Bäumer et al. and Yasui et al. reported that the lateral penumbra width differences were within ±0.5 mm and ±1.5 mm with spot scanning systems using a patient‐specific aperture,^11^, ^31^ respectively. Vilches‐Freixas et al. reported that the lateral penumbra agreements for both the left and right sides were within ±1.5 mm with the adaptive aperture PBS system.^29^ Our results were more consistent with those of the penumbra than their reports, except for Bäumer's results, achieving accurate dose calculations and beam measurements. However, as Vilches‐Freixas also reported, the collimator attached to a treatment nozzle such as the MLC may cause leaf position errors when moving.^32^ To treat the PBS with the MLC accurately and safely, we need to perform periodic detailed machine quality assurance of collimator position accuracy. We will evaluate additional detailed verification of the combined robustness of the beam spot position variation and collimator position variation.^35^

Our study has explored the prospects and several limitations of TPS validations. First, only one collimator opening pattern dose distribution was verified for each target volume. As the spatial distance between the MLC and CTV reduces, the spot region and MLC overlap are expected to increase to generate a large number of collimated secondary protons, which may affect the dose calculations. In treatment planning situations, the collimator opening is freely determined for each treatment site. Therefore, we need to investigate the agreements between the calculations and measurements of the plans in which the spatial distance between the target and the MLC is different from the set value in the current study. Second, the dose verification has been performed at one type of nozzle position only. A shorter beam distance between the MLC and the patient (closer air gap) might lead to the dose change in the shallow region with scattered protons from the MLC. In addition, the beam with a larger air gap with RS was overestimated by the effect of the nuclear halo.^13^ This effect may cause a dose discrepancy between the measurements and the calculations. Although inserting the MLC into such a beam may increase the difficulty of dose calculation reproducibility, further consideration should be given to the calculation accuracy.

Our new PBS machine expected that collimated plans could improve lateral penumbra more than uncollimated plans. However, we still do not understand the characteristics of the PBS plans with the MLC in detail. In the future, we also plan to investigate several scenarios as shown below: (1) the robustness relationship between the collimated and uncollimated plans, (2) the changes in penumbra size for varying beam parameters, and (3) the development of a dynamic collimation PBS delivery technique.^32^, ^36^, ^37^, ^38^ First, in the uncollimated plans, the worst‐case optimization intentionally creates a gentle dose gradient in the penumbra region. Thus, high‐energy plans with a small beam size reduce the physical advantage of providing a sharper penumbra. In contrast, the collimated plans change the gentle dose gradient to a sharp gradient again, and unacceptable target coverage degradation may be observed if lateral setup errors occur. We will investigate the relationship between the collimator margins and target robustness. Next, several factors of changing penumbra are considered, such as spot placements, air gaps, beam sizes (depth), MLC margins, and the presence or absence of RS. In this study, we verified the dose agreements between the calculations and measurements using only one type of beam parameter. Maes et al. proposed the formulation of the penumbra width for various beam parameters in a PBS plan with a patient‐specific collimator and predicted the dose advantage of the collimated plans.^36^ Our systems could also improve the quality of treatment by conducting such studies. Furthermore, the advantage of the collimated beam with MLC is that it can be irradiated while changing the position of each energy layer. To validate in advance due to the future software/hardware update, the TPS can calculate the dynamic collimation fields by the in‐house program. Smith et al. compared three types of PBS plans for five brain tumors using dynamic collimation, static collimated, and uncollimated plans.^37^ They concluded that the dynamic collimation plans could reduce the OAR doses more than the static collimated plans. We expect that the clinical use of our dynamic collimation system will provide even better proton therapy.

## 5 CONCLUSION

We have verified the PBS plans with the MLC calculated by the commercial MC dose engine. The commissioning results were consistent between the measurements and TPS calculations in terms of the DDs, LP, and ADs. However, the γ‐passing deteriorated in several LP measurements in less than 75 mm regions. The dose calculation errors were due to the potential limitations of beam scattering from the MLC and the beam modeling of the TPS. The discrepancies in dose verifications in shallow regions can be mitigated by interventions such as using uncollimated beams or changing treatment angles. Further validations are also required because the results of this study do not cover all the commissioning of TPS in the available PBS beam conditions. To understand the characteristics of the PBS plans with the MLC in detail, we will investigate and develop of the MLC dynamic collimation plans.

## AUTHOR CONTRIBUTIONS

Yuki Tominaga, Yusuke Sakurai, Junya Miyata, and Masataka Oita conceived and designed the study, collected the data, contributed data or analysis tools, performed the analysis, wrote the paper, and revised the paper. Shuichi Harada and Takashi Akagi provided clinical expertise and supervision of the project.

## CONFLICT OF INTEREST

The authors declare that they have no conflicts of interest.

## References

[1] Van De Water TA , Lomax AJ , Bijl HP , et al. Potential benefits of scanned intensity‐modulated proton therapy versus advanced photon therapy with regard to sparing of the salivary glands in oropharyngeal cancer. Int J Radiat Oncol Biol Phys. 2011;79(4):1216‐1224. 20732761 10.1016/j.ijrobp.2010.05.012

[2] Pedroni E , Bacher R , Blattmann H , et al. The 200‐MeV proton therapy project at the Paul Scherrer Institute: conceptual design and practical realization. Med Phys. 1995;22(1):37‐53. 7715569 10.1118/1.597522

[3] Zhu XR , Poenisch F , Lii M , et al. Commissioning dose computation models for spot scanning proton beams in water for a commercially available treatment planning system. Med Phys. 2013;40(4):041723. 23556893 10.1118/1.4798229 PMC3631269

[4] Zhang X , Li Y , Pan X , et al. Intensity‐modulated proton therapy reduces the dose to normal tissue compared with intensity‐modulated radiation therapy or passive scattering proton therapy and enables individualized radical radiotherapy for extensive stage iiib non‐small‐cell lung cancer: a virtual clinical study. Int J Radiat Oncol Biol Phys. 2010;77(2):357‐366. 19660879 10.1016/j.ijrobp.2009.04.028 PMC2868090

[5] Farr JB , Mascia AE , his WC , et al. Clinical characterization of a proton beam continuous uniform scanning system with dose layer stacking. Med Phys. 2008;35(11):4945‐4954. 19070228 10.1118/1.2982248 PMC2673594

[6] Matsuura T , Fujii Y , Takao S , et al. Development and evaluation of a short‐range applicator for treating superficial moving tumors with respiratory‐gated spot‐scanning proton therapy using real‐time image guidance. Phys Med Biol. 2016;61(4):1515‐1531. 26815927 10.1088/0031-9155/61/4/1515

[7] Shirey RJ , Wu HT . Quantifying the effect of air gap, depth, and range shifter thickness on TPS dosimetric accuracy in superficial PBS proton therapy. J Appl Clin Med Phys. 2018;19(1):164‐173. 29239528 10.1002/acm2.12241 PMC5768007

[8] Carabe A , Moteabbed M , Depauw N , Schuemann J , Paganetti H . Range uncertainty in proton therapy due to variable biological effectiveness. Phys Med Biol. 2012;57(5):1159‐1172. 22330133 10.1088/0031-9155/57/5/1159

[9] Meier G , Leiser D , Besson R , et al. Contour scanning for penumbra improvement in pencil beam scanned proton therapy. Phys Med Biol. 2017;62(6):2398‐2416. 28151727 10.1088/1361-6560/aa5dde

[10] Kralik JC , Xi L , Solberg TD , Simone CB , Lin L . Comparing proton treatment plans of pediatric brain tumors in two pencil beam scanning nozzles with different spot sizes. J Appl Clin Med Phys. 2015;16(6):41‐50. 26699553 10.1120/jacmp.v16i6.5389 PMC5690992

[11] Yasui K , Toshito T , Omachi C , et al. A patient‐specific aperture system with an energy absorber for spot scanning proton beams: verification for clinical application. Med Phys. 2015;42(12):6999‐7010. 26632055 10.1118/1.4935528

[12] Yasui K , Toshito T , Omachi C , et al. Evaluation of dosimetric advantages of using patient‐specific aperture system with intensity‐modulated proton therapy for the shallow depth tumor. J Appl Clin Med Phys. 2018;19(1):132‐137. 29178546 10.1002/acm2.12231 PMC5768032

[13] Saini J , Maes D , Egan A , et al. Dosimetric evaluation of a commercial proton spot scanning Monte‐Carlo dose algorithm: comparisons against measurements and simulations. Phys Med Biol. 2017;62(19):7659‐7681. 28749373 10.1088/1361-6560/aa82a5

[14] Fukumitsu N , Yamashita T , Mima M , Demizu Y , Suzuki T , Soejima T . Dose distribution effects of spot‐scanning proton beam therapy equipped with a multi‐leaf collimator for pediatric brain tumors. Oncol Lett. 2021;22(2):635. 34295382 10.3892/ol.2021.12896 PMC8273856

[15] Sugiyama S , Katsui K , Tominaga Y , et al. Dose distribution of intensity‐modulated proton therapy with and without a multi‐leaf collimator for the treatment of maxillary sinus cancer: a comparative effectiveness study. Radiat Oncol. 2019;14(1):209. 31752928 10.1186/s13014-019-1405-y PMC6873663

[16] Schreuder AN , Bridges DS , Rigsby L , et al. Validation of the RayStation Monte Carlo dose calculation algorithm using realistic animal tissue phantoms. J Appl Clin Med Phys. 2019;20(10):160‐171. 10.1002/acm2.12733 PMC6806482 31541536

[17] Schreuder AN , Bridges DS , Rigsby L , et al. Validation of the RayStation Monte Carlo dose calculation algorithm using a realistic lung phantom. J Appl Clin Med Phys. 2019;20(12):127‐137. 10.1002/acm2.12777 PMC6909115 31763759

[18] Winterhalter C , Lomax A , Oxley D , Weber DC , Safai S . A study of lateral fall‐off (penumbra) optimisation for pencil beam scanning (PBS) proton therapy. Phys Med Biol. 2018;63(2):025022. 29324441 10.1088/1361-6560/aaa2ad

[19] Winterhalter C , Meier G , Oxley D , Weber DC , Lomax AJ , Safai S . Contour scanning, multi‐leaf collimation and the combination thereof for proton pencil beam scanning. Phys Med Biol. 2019;64(1):015002. 10.1088/1361-6560/aaf2e8 30523928

[20] Kim DH , Park S , Jo K , et al. Investigations of line scanning proton therapy with dynamic multi‐leaf collimator. Phys Medica. 2018;55:47‐55. 10.1016/j.ejmp.2018.10.009 30471819

[21] RaySearch Laboratory. RayStation 9A reference manual. 2019.

[22] Soukup M , Fippel M , Alber M . A pencil beam algorithm for intensity modulated proton therapy derived from Monte Carlo simulations. Phys Med Biol. 2005;50(21):5089‐5104. 16237243 10.1088/0031-9155/50/21/010

[23] Pedroni E , Scheib S , Böhringer T , et al. Experimental characterization and physical modelling of the dose distribution of scanned proton pencil beams. Phys Med Biol. 2005;50(3):541‐561. 15773729 10.1088/0031-9155/50/3/011

[24] Ezzell GA , Burmeister JW , Dogan N , et al. IMRT commissioning: multiple institution planning and dosimetry comparisons, a report from AAPM Task Group 119. Med Phys. 2009;36(11):5359‐5373. 19994544 10.1118/1.3238104

[25] Fredriksson A , Forsgren A , Hårdemark B . Minimax optimization for handling range and setup uncertainties in proton therapy. Med Phys. 2011;38(3):1672‐1684. 21520880 10.1118/1.3556559

[26] Liu W , Zhang X , Li Y , Mohan R . Robust optimization of intensity modulated proton therapy. Med Phys. 2012;39(2):1079‐1091. 22320818 10.1118/1.3679340 PMC3281975

[27] Hong L , Goitein M , Bucciolini M , et al. A pencil beam algorithm for proton dose calculations. Phys Med Biol. 1996;41(8):1305‐1330. 8858722 10.1088/0031-9155/41/8/005

[28] Low DA , Harms WB , Mutic S , Purdy JA . A technique for the quantitative evaluation of dose distributions. Med Phys. 1998;25(5):656‐661. 9608475 10.1118/1.598248

[29] Charlwood FC , Aitkenhead AH , MacKay RI . A Monte Carlo study on the collimation of pencil beam scanning proton therapy beams. Med Phys. 2016;43(3):1462‐1472. 26936730 10.1118/1.4941957

[30] Farr JB , Moyers MF , Allgower CE , et al. Clinical commissioning of intensity‐modulated proton therapy systems: report of AAPM task group 185. Med Phys. 2021;48(1):e1‐e30. 33078858 10.1002/mp.14546

[31] Baumer C , Janson M , Timmermann B , Wulff J . Collimated proton pencil‐beam scanning for superficial targets: impact of the order of range shifter and aperture. Phys Med Biol. 2018;63(8):085020. 29553047 10.1088/1361-6560/aab79c

[32] Vilches‐Freixas G , Unipan M , Rinaldi I , et al. Beam commissioning of the first compact proton therapy system with spot scanning and dynamic field collimation. Br J Radiol. 2020;93(1107):20190598. 31782941 10.1259/bjr.20190598 PMC7066978

[33] Kang M , Pang D . Commissioning and beam characterization of the first gantry‐mounted accelerator pencil beam scanning proton system. Med Phys. 2020;47(8):3496‐3510. 31840264 10.1002/mp.13972

[34] Saini J , Cao N , Bowen SR , et al. Clinical commissioning of a pencil beam scanning treatment planning system for proton therapy. Int J Part Ther. 2016;3(1):51‐60. 31772975 10.14338/IJPT-16-0000.1 PMC6871575

[35] Li H , Sahoo N , Poenisch F , et al. Use of treatment log files in spot scanning proton therapy as part of patient‐specific quality assurance. Med Phys. 2013;40(2):021703. 23387726 10.1118/1.4773312 PMC3555925

[36] Maes D , Regmi R , Taddei P , et al. Parametric characterization of penumbra reduction for aperture‐Parametric characterization of penumbra reduction for aperture‐collimated pencil beam scanning (PBS) proton therapy. Biomed Phys Eng Express. 2019;5(3):035002.

[37] Smith B , Gelover E , Moignier A , et al. Technical note: a treatment plan comparison between dynamic collimation and a fixed aperture during spot scanning proton therapy for brain treatment. Med Phys. 2016;43(8):4693‐4699. 27487886 10.1118/1.4955117 PMC5360163

[38] Grewal HS , Ahmad S , Jin H . Performance evaluation of adaptive aperture's static and dynamic collimation in a compact pencil beam scanning proton therapy system: a dosimetric comparison study for multiple disease sites. Med Dosim. 2021;46(2):179‐187. 33279369 10.1016/j.meddos.2020.11.001

